# Human papillomavirus in head and neck squamous cell carcinomas in a South African cohort

**DOI:** 10.1016/j.pvr.2018.10.006

**Published:** 2018-11-01

**Authors:** Tumelo R. Sekee, Felicity J. Burt, Dominique Goedhals, Jacqueline Goedhals, Yuri Munsamy, Riaz Y. Seedat

**Affiliations:** a Division of Virology, Faculty of Health Sciences, University of the Free State, Bloemfontein, South Africa; b Division of Virology, National Health Laboratory Service Universitas, Bloemfontein, South Africa; c Department of Anatomical Pathology, Faculty of Health Sciences, University of the Free State, Bloemfontein, South Africa; d Department of Anatomical Pathology, National Health Laboratory Service Universitas, Bloemfontein, South Africa; e Department of Otorhinolaryngology, Faculty of Health Sciences, University of the Free State, Bloemfontein, South Africa; f Department of Otorhinolaryngology, Universitas Academic Hospital, Bloemfontein, South Africa

**Keywords:** Human papillomavirus, Oropharyngeal carcinoma, Laryngeal carcinoma, Hypopharyngeal carcinoma, Nasopharyngeal carcinoma

## Abstract

**Background:**

Most tumours of the head and neck are attributable to smoking and alcohol use, but an increasing proportion of head and neck tumours are caused by human papillomaviruses (HPVs). The aim of this study was to use in house molecular assays to detect and genotype HPV in biopsies from patients with histologically confirmed head and neck squamous cell carcinomas. In addition, the results were compared with p16 immunohistochemistry staining, which has been described as a potential marker for HPV infection.

**Methods:**

Biopsies of squamous cell carcinomas of the oropharynx, nasopharynx, larynx and hypopharynx from 112 South African patients were screened using three PCR assays targeting the L1 and E6 regions of HPV and p16 immunohistochemical staining.

**Results and conclusion:**

HPV was identified in 7 (6.3%) tumours, while 22 (19.6%) had positive p16 immunohistochemical staining. There was concordance between the results obtained using the three PCR assays. There was substantial agreement between the results of molecular tests and p16 immunohistochemistry for hypopharyngeal carcinomas, but only fair agreement for laryngeal and oropharyngeal carcinomas.

## 1 Introduction

Head and neck tumours are the sixth most common group of tumours worldwide [1]. Squamous cell carcinomas (SCCs) of the head and neck can be attributed to smoking and alcohol use; however an increasing proportion of head and neck SCCs are caused by human papillomaviruses (HPVs), with HPV now being the primary cause of tonsillar carcinoma in North America and Northern Europe [1]. Globally, approximately 38,000 cases of head and neck SCCs are attributable to HPV [2]. Countries located in North America and Europe have a relatively high (over 1.25 per 100,000) age-standardised incidence rate of HPV-attributable head and neck cancer [2]. Data on HPV prevalence in sub-Saharan Africa is limited and based on application of a variety of molecular or histological assays [3], [4], [5], [6], [7], [8], [9], [10], [11]. The prognosis of patients with HPV-positive oropharyngeal carcinoma is more favourable than that of patients with HPV-negative tumours, making the detection of HPV an important biomarker for predicting patient outcome [12].

HPV infections are diagnosed using molecular techniques as the virus cannot be cultured and serology has a limited accuracy [13]. Although commercial assays are available, these assays are designed and validated for HPV detection in cervical samples. Commercial assays are costly for performing surveillance, and we estimate that commercial assays would cost almost three times more than in-house assays. Therefore in-house assays are advantageous from a cost perspective. It was deemed appropriate to compare in-house assays, commercial assays and p16 immunostaining, a proposed surrogate marker of HPV transformation. This comparison was to identify an assay for use in long-term surveillance. Assays using primers targeting the L1 gene have previously been used for the detection of HPV in head and neck tumours. However integration of the virus may result in loss of large regions of the genome, most often involving E2 and E1, but also L1 and L2, resulting in failure to detect the virus using these primers [11]. The E6 region is the least conserved between different HPV types; however it is always preserved, even in the event of viral integration [14]. Hence an in-house assay was developed targeting the E6 region, taking advantage of the heterogeneity between types to develop type-specific assays.

The aim of this study was to use in-house molecular assays to detect and genotype HPV in biopsies from patients with histologically confirmed head and neck SCCs.

## 2 Materials and methods

### 2.1 Samples and DNA extraction

A total of 112 patients admitted at the Universitas Academic Hospital in Bloemfontein, South Africa between January 2014 and July 2017 with histologically confirmed SCCs of the hypopharynx (n = 10), oropharynx (n = 20) (including 8 tonsillar carcinomas), nasopharynx (n = 3), and larynx (n = 79) were enrolled in the study. The study was approved by the Health Sciences Research Ethics Committee, University of the Free State (137/2013B). Written informed consent was obtained from each patient.

DNA was extracted from freshly frozen tissue biopsies using QIAamp DNA Mini Kit from QIAGEN according to manufacturer's instructions (QIAGEN, Valencia, CA, USA). Tissue pieces were approximately three to four millimetres in diameter and DNA was eluted in a final volume of 200 µl AE buffer supplied in the kit and stored at −80 °C.

### 2.2 Polymerase chain reaction methods

Three molecular methods, which included two nested PCR assays targeting the L1 region and an in-house multiplex hemi-nested PCR using type-specific primers that target the E6 gene, were used to screen tissue biopsies for HPV DNA.

#### 2.2.1 Nested PCR, MY09/11 and GP5+/6+ primer pairs

A nested PCR was performed using MY09/11 as outer primers and GP5+/6+ as inner primers [15], [16]. These primer pairs amplify a 450 bp (MY09/11) and 140 bp (GP5+/6+) region of the HPV L1 gene. PCR reactions were performed using GoTaq®flexi kit (Promega, Madison, USA) according to manufacturers’ instructions using 5 µl of extracted DNA, 0.4 µM of forward and reverse primers. The PCR reaction was cycled using the following conditions: initial denaturation, 95 °C for two minutes followed by 30 cycles of denaturation at 95 °C for 30 s, annealing temperature at 47 °C for 30 s and elongation at 72 °C for one minute and final elongation at 72 °C for five minutes. Samples that were negative were further amplified using GP5+/6+ nested primer set and GoTaq®flexi kit as previously described but with an annealing temperature of 43 °C.

The beta-globin gene was amplified concurrently as a control for DNA integrity using PCO4 and GH20 primer pair [17]. All PCR reactions were separated and visualised on 1% agarose gels stained with ethidium bromide.

#### 2.2.2 Nested PCR, PGMY09/11 and GP5 + /6 + primer pairs

HPV DNA was amplified using modified PGMY09/11 and GP5+/GP6+ [18]. This modified primer set includes nine primer pairs that were designed to increase the specificity of the original consensus primers. PCR reactions were performed as described above (2.2.1) using an annealing temperature of 55 °C for the first round of amplification.

#### 2.2.3 E6 multiplex hemi-nested type specific PCR

A multiplex PCR reaction was developed using primers that target the E6 gene of low risk HPV types 6 and 11 and high risk HPV types 16, 18, 31, 33, 45 and 58. Primers were designed based on alignment of DNA sequences retrieved from GenBank for HPV types 6, 11, 16, 18, 31, 33, 45 and 58. Each primer sequence and the HPV type targeted is listed in Table 1, Table 2.

**Table 1:** Nucleotide sequence of primers targeting a region of the E6 gene identified for first round PCR amplification.

**Primer**^a^	**Nucleotide sequence (5′to 3′ direction)**	**Amplicon size (bp)**	**Nucleotide position**^b^
HPV6F	5′CCTCCACGTCTGCAACGACCA3′	174 bp	837–858
HPV6R	5′AGGCTGCATATGGATAGCCGG3′		991–1010
HPV11F	5′ATGGAAAGTAAAGATGCCTCCACGT3′	200 bp	102–126
HPV11R	5′CAACAGGCACACGCTGCAAG3′		281–300
HPV16F	5′AGGACCCACAGGAGCGAC3′	147 bp	111–128
HPV16R	5′TGCATAAATCCCGAAAAGCAAAGTC 3′		233–257
HPV18F	5′ATGGCGCGCTTTGAGGATCC3′	191 bp	105–125
HPV18R	5′GCAGCATGCGGTATACTGTCT3′		275–295
HPV31F	5′CGGCATTGGAAATACCCTACGA 3′	141 bp	157–178
HPV31R	5′GCACACACTCCGTGTGGTGTG3′		278–298
HPV33F	5′ GAGAGGGAAATCCATTTGGAATATG3′	178 bp	164–188
HPV33R	5′TCTTGAGGACACAAAGGTCTTTG3′		319–341
HPV45F	5′GGCGCGCTTTGACGATCCAAAG3′	136 bp	03–24
HPV45R	5′TTGATATACCTCTGTGCGTTCC3′		117–138
HPV58F	5′ATGTTCCAGGCACAGAGGAGAAAC3′	196 bp	110–135
HPV58R	5′ CACTTTACATACTGCAAATGGATTC3′		281–306

a Number indicates HPV type.

b Nucleotide positions relative to isolates retrieved from GenBank (GenBank accession numbers: HPV6 - JN573163.1, HPV11 - M14119.1, HPV16 - AF125673.1, HPV18 - AY262282.1, HPV31 - KU298890.1, HPV33 - KC662563.1, HPV45 - KC662571.1, HPV58 - KU298920.1)

**Table 2:** Nucleotide sequence of forward primers targeting a region of the E6 gene identified for use in hemi-nested PCR reaction.

**Primer**^a^	**Nucleotide sequences (5′to 3′ direction)**	**Amplicon size (bp)**	**Nucleotide position**^b^
HPV6F2	5′GCAAGAATGCACTGACCACTGCAG3′	90 bp	921–944
HPV11F2	5′CTTTGCACACTCTGCAAATTCAG3′	133 bp	169–181
HPV16F2	5′CCACAGTTATGCACAGAGCTGCAA3′	117 bp	139–163
HPV18F2	5′GTGCACGGAACTGAACACTTCACT3′	141 bp	155–178
HPV31F2	5′CTGCAAAGGTCAGTTAACAGAAAC3′	96 bp	203–226
HPV33F2	5′CTGTGTTTGCGGTTTTTATCTAAAC3′	149 bp	193–217
HPV45F2	5′CCCTACAAGCTACCAGATTTG3′	107 bp	31–51
HPV58F2	5′GTCAGGCGTTGGAGACATCTGTGC3′	149 bp	157–181

a Number indicates HPV type.

b Nucleotide positions relative to isolates retrieved from GenBank (GenBank accession numbers: HPV6 - JN573163.1, HPV11 - M14119.1, HPV16 - AF125673.1, HPV18 - AY262282.1, HPV31 - KU298890.1, HPV33 - KC662563.1, HPV45 - KC662571.1, HPV58 - KU298920.1).

The low and high-risk PCR assays were performed separately, to prevent primer binding competition, with an annealing temperature of 59 °C. One microliter of the first round PCR reaction was used as the template for the hemi-nested PCR. PCR reactions were separated and visualised on 2.5% agarose gels stained with ethidium bromide.

#### 2.2.4 Roche Linear Array® HPV genotyping test

The Roche Linear Array HPV Genotyping Test (Roche Molecular Systems, New Jersey, USA) was performed according to manufacturer's instructions on the first 74 samples submitted. The remaining samples were not tested due to financial constraints. Briefly, the PCR was performed using primers provided in the kit. The 100 µl reaction comprised of 50 µl master mix containing MgCl_2_, Amplitaq Gold DNA polymerase (Roche Molecular Systems, New Jersey, USA), uracil-N-glycosilase, deoxynucleotides, biotinylated primers and 50 µl of DNA template. An internal control was incorporated in the assay with the inclusion of primers to amplify the beta-globin gene. The reaction was cycled as follows: 50 °C for 2 min, 95 °C for 9 min and 40 cycles of 95 °C for 30 s, 55 °C for 1 min, 72 °C for 1 min and finally, at 72 °C for 5 min before holding it indefinitely at 72 °C.

The amplicons were denatured and hybridised to a strip containing specific probes for 37 HPV genotypes and the beta-globin reference. Colorimetric determination was performed using the linear array detection kit. Positive reactions appeared as blue lines on the strip. The strips were interpreted using the HPV reference guide provided.

### 2.3 HPV genotyping

PCR products were excised from the 1% or 2.5% agarose gels and were purified using Wizard®SV Gel and PCR Clean-Up Systems according to manufacturer's instructions (Promega, Wisconsin, USA). Determination of nucleotide sequence of the amplicon was performed using the Big Dye Terminator sequencing ready reaction kit according to manufacturer's instructions (Applied Biosystems, Foster City, CA, USA). Purified PCR amplicons were sequenced bidirectionally. Nucleotide sequences obtained for PCR positive amplicons were edited using Chromas Pro version 1.6 and aligned using Clustal Omega 1.2.1 [19]. HPV genotypes were determined by comparison with nucleotide sequence data retrieved from GenBank and BLAST analysis.

### 2.4 Controls

HPV11 DNA extracted in an unrelated study from a patient with confirmed recurrent respiratory papillomatosis was used as a positive control for low risk HPV types in PCR reactions targeting the L1 gene. HPV16 DNA was used as a positive control for high risk HPV types. The PCR targeting the L1 gene is well established and known to amplify multiple HPV types. The specificity of the in house PCR targeting the E6 gene was confirmed using HPV16, HPV18, HPV31 and HPV45 DNA extracted from patients. In the absence of clinical controls for HPV33 and HPV58, DNA controls were prepared by using gene fragments synthesized by GenScript (Piscataway, NJ, USA) and supplied in pUC 57 plasmid. DNA template for use as a positive control was prepared by amplifying DNA from the plasmid using a type specific forward primer and M13 primer targeting the plasmid. Amplicons were gel purified and diluted for use as DNA template.

### 2.5 p16 staining

Tissue sections of 4 µm were cut from wax blocks of formalin fixed paraffin embedded specimens and stained using the CINtec®-p16 kit (Ventana Medical Systems, Inc., Tucson, USA) according to the manufacturer's instructions. All stains were performed on a Benchmark XT automated slide preparation system (Ventana Medical Systems Inc., Tucson, USA) using diaminobenzidene as a chromogen. The p16 expression was reported as positive if at least 70% of the tumour cells showed moderate to strong nuclear and cytoplasmic staining.

### 2.6 Statistical analysis

Statistical analysis was performed with IBM SPSS version 25. Differences between ages were analysed using the independent samples *t*-test, while associations were analysed with Fisher's exact test. Agreement between the results of molecular tests and p16 immunohistochemistry was performed using Cohen's kappa and classified according to the criteria of Landis and Koch [20].

## 3 Results

There were 102 (91.1%) males and 10 (8.9%) females. The average age of the patients was 61.5 years (range 37.3–85.2 years). The mean age for males was 61.5 years (range 37.3–85.2 years, median 60.9 years) and for females was 62.1 years (range 55.0–71.0 years, median 61.2 years), with no significant difference in age between males and females (p = 0.846).

Amplification of the beta-globin gene from all samples confirmed the viability of extracted DNA. The MY09/11 and GP5+/6+ primers amplified HPV DNA from 6/112 (5.4%) samples. HPV types 11, 16, 18, and 31 were detected (Table 3). The PGMY09/11 and GP5+/6+ primers detected HPV DNA in 7/112 (6.3%) samples (the same 6 samples in which the MY09/11 and GP5+/6+ primers were positive and an additional sample that was positive for HPV45). The multiplex hemi-nested PCR targeting the E6 gene detected HPV DNA in the same 7/112 (6.3%) samples. An overall total of 7/112 (6.3%) samples tested positive for HPV DNA using the three in-house assays and genotypes were confirmed by sequencing (Table 3).

**Table 3:** HPV types detected per site.

**Site**	**HPV types**
Hypopharynx	11
Larynx	11, 16, 31, 45
Oropharynx	16
Nasopharynx	18
**Total positive/tested**	**7/112**

The Roche Linear Array HPV Genotyping Test was performed on the first 74 samples, five of which were positive using the PCR assays. Of the five positive samples, three tested positive for HPV types 16, 18 and 45. These were the same HPV types detected with the PCR assays. The two positive samples that were not detected using the linear array were an HPV11 and HPV31. The linear array did not detect HPV in any of the samples that tested negative for HPV using the PCR assays.

There was no significant difference in the age of patients with HPV positive tumours (mean 58.6 years, range 37.3–74.2 years) and HPV negative tumours (mean 61.7 years, range 42.0–85.2 years) (p = 0.620). There was no association between gender and HPV positivity (p = 0.119).

Twenty-two (19.6%) samples had positive p16 stains (Table 4). For the entire group, there was moderate agreement between the presence of HPV DNA and a positive p16 stain (κ = 0.407, p = 0.000). There was substantial agreement between the results of molecular tests and p16 immunohistochemistry for hypopharyngeal carcinomas, but only fair agreement for laryngeal and oropharyngeal carcinomas (Table 4). All samples that were HPV positive, except for the HPV11 isolate from the laryngeal carcinoma, were also p16 positive. HPV DNA was not detected in this isolate with the linear array, but was detected with all the in-house assays. The p16 immunostain was also positive on the sample in which HPV31 was detected using the in-house assays but negative with the linear array, as well as the sample in which HPV45 was not detected with the MY09/11 and GP5+/6+ primers but detected with the other assays.

**Table 4:** Summary of PCR and p16 findings.

**Sites**	**HPV detected positive/total number of samples**	**p16 positive positive/total**	**K value**
Hypopharynx	1/10 (10%)	2/10	κ = 0.615, p = 0.035
Larynx	4/79 (5.06%)	11/79	κ = 0.352, p = 0.000
Oropharynx	1/20 (5.0%)	5/20	κ = 0.273, p = 0.076
Nasopharynx	1/3 (33.3%)	1/3 (33.33%)	κ = 1.00, p = 0.083
**Total**	**7/112 (6.25%)**	**19/112 (16.96%)**	**N/A**

*N/A-not applicable.

## 4 Discussion

HPV was identified in 7 of 112 (6.3%) SCCs, including tumours of the oropharynx, nasopharynx, hypopharynx and larynx. Six of these were high risk types.

Three molecular assays were compared, namely two nested PCR assays targeting the L1 gene and a hemi-nested PCR targeting the E6 gene. Nested PCR using MY09/11 and GP5+/6+ detected 6/112 (5.4%) HPV types; nested PCR using PGMY 09/11 and GP5+/6+ detected 7/112 (6.3%) HPV types and the hemi-nested PCR targeting the E6 gene detected 7/112 (6.3%). Three of the five samples that tested positive for HPV DNA using the three in-house molecular assays were positive when tested using the commercial linear array while the remaining two samples were negative. These results were unexpected as genotyping confirmed that the samples were HPV11 and HPV31, which are types included in the linear array. As the assay is designed to detect HPV11 and HPV31 it could possibly be surmised that the negative results may be due to a low viral load. Previous reports on viral loads in head and neck tissues have suggested that the viral load can vary and this is an aspect that requires further investigation [21]. The impact of viral load on disease outcome is unknown.

The p16 stain is often used as a surrogate marker for HPV detection as inactivation of the pRB by HPV E7 induces p16 upregulation [22]. However the p16 stain is not a reliable marker for the presence of HPV in carcinomas with a low prevalence of HPV because alternative mechanisms can induce p16 overexpression [23]. We found that 10.1% of laryngeal tumours and 20% of oropharyngeal tumours were p16 positive despite being HPV negative, supporting this view. In these patients, p16 overexpression could suggest pRB pathway disturbances unrelated to HPV [1].

There was moderate agreement overall between the results of p16 immunohistochemistry and detection of HPV DNA. Bussu et al. found a poor agreement between p16 immunohistochemistry and the detection of HPV RNA in head and neck SCCs, except for tumours of the oropharynx, where there was a fair agreement [24]. Dale et al. found that in oropharyngeal carcinoma, although p16 immunohistochemistry had a negative predictive value of 100%, it had a positive predictive value of 57% [12].

HPV-negative p16-positive laryngeal tumours may be associated with a worse prognosis [23], while in oropharyngeal tumours, the survival rate of patients with HPV-negative p16-positive tumours is similar to that of patients with HPV-negative tumours [25], [26].

The proportion of HPV positive head and neck tumours varies worldwide [2]. The HPV attributable fraction of oropharyngeal tumours is over 40% in more developed countries (Europe, Northern America, Australia, New Zealand, Japan and Republic of Korea), but much lower in Africa and Latin America [2]. However, in oral cavity and laryngeal carcinomas, the prevalence of HPV is lower (1.4–4.6%) and more homogeneous across countries [2].

Most studies from sub-Saharan Africa have found a low prevalence of HPV in head and neck SCCs [2], [3], [4], [5], [6], [7], [8], [9], [10]. However the differences in prevalence that have been previously reported may be result of using different techniques with different sensitivities. Earlier studies relied on immunohistochemistry and in situ hybridisation assays which lack the sensitivity of molecular assay that can amplify low viral loads to demonstrable levels [3]. HPV was not demonstrated in 51 formalin-fixed paraffin embedded (FFPE) samples (22 oropharyngeal and 29 oral tongue SCCs) tested using immunohistochemistry and HPV 16 E7/E7 type specific PCR [9]. Similarly 149 FFPE head and neck tumours (excluding tumours of the oropharynx) tested for HPV DNA using Roche linear array were negative [10]. In contrast, a Ghanaian study of 78 FFPE head and neck SCCs using a multiplex PCR targeting the E6 region found an overall HPV prevalence of 19.23%, with HPV identified in 24.2% of laryngeal tumours and 18.2% of pharyngeal tumours, although it was not identified in any tonsillar tumours [8]. A recent South African study on oropharyngeal carcinoma using PCR targeting the L1, E5, E6/E7 regions, in situ hybridisation and immunohistochemistry on formalin-fixed, paraffin- embedded samples found that 94.1% of 51 oropharyngeal tumours were positive for HPV DNA of which 49.1% were HPV-driven (positive for HPV by PCR as well as p16 positive) [11]. Of the HPV-driven tumours, the most common genotypes identified were: HPV16 and HPV31 co-infection (32.0%), HPV16 (32.0%), HPV31 (24.0%), HPV16 and HPV18 co-infection (8.0%), and HPV18 (4.0%). In this study, HPV DNA was amplified from one of 20 (5.0%) oropharyngeal carcinomas that was also p16 positive, suggesting that it was HPV-driven. Tumour p16 and HPV DNA positivity have been shown to be biomarkers for improved survival in oropharyngeal squamous cell carcinoma [12], [22], [27].

Four of the 79 (5.1%) laryngeal carcinomas were HPV positive, 3 for high risk types (HPV16, HPV31 and HPV45) and one for a low risk type (HPV11). All three tumours that were positive for high risk types were p16 positive. Hernandez et al. found HPV DNA in 21% of laryngeal carcinomas with a limited correlation with p16 status [28]. HPV and/or p16 positivity was not found to be a predictor of survival.

One of the three nasopharyngeal carcinomas was positive both for HPV18 and p16 and EBER (Epstein-Barr encoding region) immunohistochemistry negative. It has been suggested that HPV may play a role in non-EBV-related nasopharyngeal carcinoma [22]. Studies on a larger group of nasopharyngeal carcinomas in South African patients are required to determine the role of HPV in these tumours.

## 5 Conclusion

Overall there was an HPV prevalence rate of 6.3% in 112 head and neck squamous cell carcinomas in a cohort of South African patients. There was only a moderate agreement between HPV DNA positivity and a positive p16 immunohistochemical stain.
